# Inter-rater reliability of the modified Sarnat examination in preterm infants at 32–36 weeks’ gestation

**DOI:** 10.1038/s41390-019-0562-x

**Published:** 2019-09-07

**Authors:** Lara Pavageau, Pablo J. Sánchez, L. Steven Brown, Lina F. Chalak

**Affiliations:** 1 Division of Neonatal-Perinatal Medicine, UT Southwestern Medical Center, Dallas, TX USA; 2 Divisions of Neonatology and Pediatric Infectious Diseases, Department of Pediatrics, Nationwide Children’s Hospital and The Ohio State University College of Medicine, Columbus, OH USA; 3 Center for Perinatal Research, The Research Institute at Nationwide Children’s Hospital, Columbus, OH USA; 4 Parkland Health & Hospital System, Dallas, TX USA

## Abstract

**Objective:**

To test the inter-rater reliability of the modified Sarnat neurologic examination in preterm neonates and to correlate abnormalities with the presence of perinatal acidosis.

**Methods:**

Prospective study of 32–36 weeks’ gestational age infants admitted to the neonatal intensive care unit. Each infant had two Sarnat examinations performed at <6 h, one by a gold standard (GS) study investigator, and the second either by (a) another GS examiner or (b) an attending physician (28 examiners), all blinded to clinical variables. Agreement was calculated using kappa (*k*) statistics.

**Results:**

One hundred and two (9, fetal acidosis) infants underwent a modified Sarnat examination. Among GS examiners, agreement was excellent (*k* > 0.8) except for Moro, while among all examiners agreement was very good (*k* > 0.7) except for both Moro and tone. Subgroup analysis at 32–34 weeks’ showed fair/poor Moro compared to excellent agreement at ≥35 weeks. Increasing abnormalities correlated with acidosis (*r* = −0.6, *P* < 0.01).

**Conclusions:**

Strong inter-rater reliability for the modified Sarnat was observed except for tone and Moro in preterm infants. Experience of the examiners resulted in improved reliability in tone, while for the Moro agreement improved only beyond 35 weeks. Findings suggest the need of adjustment of the examination form specific for preterm infants.

## Introduction

Clinical examination remains the most effective way to assess neonatal neurological status as it evaluates the integrity of the nervous system. However, the neurologic assessment of the newborn is challenging due to transient effects of delivery, need of resuscitation, general anesthesia, and/or the presence of other associated conditions, such as respiratory distress.^1,2^ These challenges are further amplified in preterm infants who can present with a complex amalgam of asphyxia and secondary maturational disturbances.^3–5^

The Sarnat stages of encephalopathy in the first week after birth was established and validated to predict neurodevelopmental outcomes following a hypoxic–ischemic injury in neonates at term.^6^ It has since been modified to allow enrollment of newborns with hypoxic–ischemic encephalopathy (HIE) in studies of neuroprotective therapies within 6 h of life. The modified Sarnat is the most commonly used neurological examination for determination of the severity of encephalopathy and eligibility for hypothermia therapy. While the modified Sarnat examination has been extensively tested in the evaluation of term newborns at risk for encephalopathy, no prior studies have tested its inter-rater reliability in preterm infants.^7^

As with any tool/method, investigation into the reliability of the modified Sarnat neurological examination performed on late and moderately preterm infants at risk for brain insult is warranted.^8,9^ Recent retrospective reports of increased prevalence of HIE in late and moderately preterm infants are difficult to interpret since the neurological examination criteria were not defined and the exam could be compounded by maturation of the nervous system.^10–12^

The objectives of this prospective study were to (1) test the inter-rater reliability of the modified Sarnat neurologic examination in late and moderately preterm neonates born at 32–36 weeks’ gestational age, and (2) correlate abnormalities of the modified Sarnat examination with the presence of perinatal acidosis.

## Methods

### Study design and patient eligibility

This prospective observational study enrolled inborn infants of 32 0/7–36 6/7 weeks’ gestation who were <6 h of age and admitted to the neonatal intensive care unit (NICU) at Parkland Health & Hospital System (PHHS) from November 2015 to February 2018. The range of the infants’ gestational age was chosen in order to determine whether there was a maturational age cutoff when the findings of the modified Sarnat neurologic examination were reliable among examiners. A study investigator (LP) identified eligible infants in the electronic health record by gestational age and time of birth. Each infant had two Sarnat examinations <6 h, one exam performed by a gold standard (GS) study investigator (L.P.), and a second exam performed, within 30 min, either by (a) a GS examiner (2 examiners) or (b) an attending neonatologist (28 examiners). All examiners were blinded to clinical/laboratory variables and the presence of perinatal acidosis or an acute perinatal event. Exclusion criteria included infants with major congenital or chromosomal anomalies or unlikely to survive for 24 h.

### Sources of data and Institutional Review Board

Pertinent demographic, clinical, laboratory, radiographic, and neuroimaging data were obtained from the electronic health records of enrolled infants and their mothers. The study was approved by the Institutional Review Board of the University of Texas Southwestern Medical Center and PHHS with a waiver of documentation of consent.

### Modified Sarnat neurologic examination

The modified Sarnat score, which was designed for infants ≥36 weeks’ gestation, contains nine items that are grouped in six categories and coded for normal/mild, moderate, and severe encephalopathy with a minimum score of 1 and maximum score of 6 (Table 1).^7^

**Table 1:** The modified Sarnat neurologic examination form utilized for the neurological assessment of all study participants

Category	Signs of HIE in each level		
	Normal/mild HIE	Moderate HIE	Severe HIE
Level of consciousness	1	2 = Lethargic	3 = Stupor/coma
Spontaneous activity	1	2 = Decreased activity	3 = No activity
Posture	1	2 = Distal flexion, complete extension	3 = Decerebrate
Tone	1	2 = Hypotonia (focal or general)	3 = Flaccid
			3 = Rigid
Primitive reflexes			
Suck	1	2 = Weak or has bite	3 = Absent
Moro	1	2 = Incomplete	3 = Absent
Autonomic system			
Pupils	1	2 = Constricted	3 = Deviation/dilated/nonreactive
Heart rate	1	2 = Bradycardia	3 = Variable HR
Respiration	1	2 = Periodic breathing	3 = Apnea or requires ventilator

*HIE* hypoxic–ischemic encephalopathy, *HR* heart rate

The neurologic examination was performed by the examiners while the infant was undressed in an open bed in a single patient room in the NICU and within the first 6 h of age.^13^ Examinations occurred in two phases consisting of observation and active manipulation.^14^ The observation phase included assessment of the arousal state, activity, posture, and heart and respiratory rates. The active phase included assessment of tone, suck reflex, Moro reflex, and pupils. Each enrolled infant had two modified Sarnat examinations performed independently, in no set order, and within 30 min of each other by the study investigator by one of the attending neonatologists and/or by one of the two GS examiners.

For the purpose of this study, a total Sarnat neurologic examination score was calculated by adding the individual score from each category: normal/mild (score = 1), moderate (score = 2), and severe (score = 3), with a total score ranging from 6 to 18.

### Examiners

Two types of examiners performed the examinations: (1) a “GS” examiner consisting of one of the three neonatologists (L.F.C. and Myra Wyckoff and the study investigator, L.P.), and (2) an attending examiner consisting of 1 of the 28 in-house neonatologists providing clinical care. The GS examiners were the most experienced as they had performed a modified Sarnat (>100 examinations) for study patients on all previous hypothermia trials.^7,15,16^ In addition, the GS examiners attended the yearly Eunice Kennedy Shriver National Institute of Health and Child Health and Development (NICHD) workshop central training meetings and certified all the remaining attending neonatologists per the institution protocol. This encompassed multiple concomitant examinations followed by a test of two matching examination forms on all items and a subsequent yearly hands on refresher and teaching lecture. Since our site was participating in the ongoing NICHD preemie 33–35 weeks’ hypothermia randomized trial,^15^ additional education was provided to all neonatologists prior to study participation and refreshed on yearly basis to incorporate preterm physiological gestational differences into the neurological examination. Therefore, all examiners were instructed that preterm tone assessment should be focused on the response to passive movement in the lower extremities, while the Moro reflex should evaluate extension of limbs, opening of hands, and extension with abduction but not flexion of the upper extremities.

### Definitions

Fetal acidemia was defined by umbilical cord gas with a pH ≤ 7.0 or base deficit (BD) ≥ −16 mmol/L, or postnatal blood gas in the first hour of age with pH ≤ 7.15 or BD ≥ −10 mmol/L and either a 10-min Apgar score ≤5 or need for assisted ventilation initiated at birth and continued for ≥10 min.^7,15,16^ Per standard care, all infants born at PHHS have an umbilical cord blood gas obtained at birth. However, if cord gas was missing, a blood gas within the first hour of birth was obtained.

HIE was diagnosed using American College of Obstetricians and Gynecologists/American Academy of Pediatrics published criteria that consisted of a combination of fetal acidosis, presence of moderate-to-severe encephalopathy affecting 3 of the 6 categories on the modified Sarnat examination, and magnetic resonance imaging brain abnormalities consistent with asphyxia, as well as multiple organ involvement.^17^

### Outcome variables

The primary objective was to test the reliability of the modified Sarnat neurologic examination in premature infants born at 32–36 weeks’ gestation. To test the inter-rater reliability, the inter-scorer agreement (kappa, *k*) was calculated (1) between the GS study investigator and the in-house neonatology attending, and (2) between the study investigator and one of the other GS examiners. A predefined subgroup analysis was performed to determine whether there was a gestational age cutoff at which the agreement was optimal overall. Secondary analyses included the correlation between the total composite exam score and umbilical cord blood gas values (pH and BD) that defined fetal acidosis.

### Statistical analysis and sample size

Statistical analysis used SPSS version 25 (IBM). Power calculation was based on the primary objective that tested agreement between two raters using kappa statistics. A minimum sample size of 85 infants achieved 80% power to detect a true kappa value of 0.80 with a significance level of 0.05. A priori decision was to enroll at least 100 infants to account for any missing data. Descriptive analyses used percentages, means, medians (25th, 75th interquartile range), and measures of variability to describe demographic data. Categorical variables were compared using Chi-square or Fisher exact test between the two cohorts of neonates, i.e., those with and without fetal acidosis. Continuous variables were evaluated using Student’s *t* test or Mann–Whitney *U* test if data were non-normally distributed.

The inter-rater agreement between examiners for each neurologic exam item was calculated using kappa intraclass correlation for each Sarnat category. Values <0.40 are considered poor, 0.40–0.59 are considered fair, 0.60–0.74 are good, and 0.75–1.0 are excellent.^18^ Analysis for each infant involved examination comparisons between (1) the GS study investigator and the in-house attending neonatologists and (2) between two GS examiners.

The subgroup analysis assessed the agreement between the neurologic examination for 32–34 weeks’ gestation and for 35–36 weeks’ gestation separately. Pearson correlation coefficient (*r*) was used to determine the correlation between the total Sarnat neurologic examination score and fetal acidosis.

## Results

Of the 1180 newborns of 32–36 weeks’ gestation admitted to the NICU during the study period, 102 (9%) were enrolled and underwent a modified Sarnat examination performed by two examiners within the first 6 h of age (mean ± SD, 3 ± 1 h), and within 20 min from each examination. Of the 102 infants, 9 (9%) had perinatal acidosis based on umbilical cord arterial blood gas values. Table 2a describes the baseline variables of infants who were admitted to the NICU with a primary diagnosis of prematurity with and without fetal acidosis. Other secondary diagnosis included respiratory distress (11 infants) and infants of diabetic mothers.^9^ The majority (73%) of mothers were Hispanic and had a cesarean delivery (73%), while most infants were male (54%) born at 32–34 weeks’ gestation (70%). The umbilical cord arterial blood gas (mean ± SD) pH was 6.97 ± 0.1 with a BD of −18 ± 7 mmol/L for newborn infants with fetal acidemia.

**Table 2a:** Clinical and laboratory characteristics of the study patients

Maternal and neonatal characteristics	Total	Fetal acidosis	No acidosis
Maternal	*N* = 80	9	71
Multiple gestation	21	0	21
Maternal age^a^	29 ± 7	33 ± 8	29 ± 7
Race/ethnicity			
White non-Hispanic	5 (6)	1 (11)	4 (6)
Black non-Hispanic	16 (20)	0	16 (23)
Hispanic	58 (73)	8 (89)	50 (70)
Asian	1 (1)	0	1 (1)
C-section	58 (73)	7 (78)	51 (72)
Pre-eclampsia	30 (38)	1 (11)	29 (41)
Diabetes	13 (16)	3 (33)	10 (14)
Perinatal event	6 (8)	2 (22)	4 (6)
Neonatal	*N* = 102	9	93
GA (weeks)^a^	34 ± 1	34 ± 1	34 ± 1
Gestational age (weeks)			
32–34	72 (70)	7 (78)	65 (70)
35	20 (20)	2 (22)	18 (19)
36	10 (10)	0	10 (11)
Female	47 (46)	5 (55)	42 (45)
Birth weight (g)*	2246 ± 514	2482 ± 884	2224 ± 465
APGAR (1 min)**	8 (6, 8)	2 (2, 4)	8 (6, 8)
APGAR (5 min)**	9 (7, 9)	6 (6, 7)	9 (7, 9)
Umbilical cord arterial blood gas^a^			
pH	7.24 ± 0.1	6.97 ± 0.1	7.26 ± 0.1
pCO_2_	58 ± 14	92 ± 22	55 ± 9
pO_2_	30 ± 3	32 ± 7	30 ± 2
Base deficit	−7 ± 5	−18 ± 7	−5 ± 3

Values in parenthesis are percentages. Data were analyzed by *t* test or *χ*^2^

*pCO*_*2*_ partial pressure of carbon dioxide, *pO*_*2*_ oxygen partial pressure

^a^Mean ± SD

^b^Median (25th, 75th interquartile range) with Mann–Whitney test

The frequency of abnormalities on each component of the modified Sarnat examination, as well as the total Sarnat score between infants with and without fetal acidosis are provided in Table 2b. Compared to infants without perinatal acidosis, those with perinatal acidosis had increased individual categories abnormalities, as well as higher total Sarnat score (12 ± 5 vs. 7 ± 2, respectively; *P* < 0.01). Seven out of nine neonates with fetal acidosis were diagnosed with moderate or severe HIE. Subsequently, five HIE patients included in this study were enrolled in the randomized controlled trial: ClinicalTrials.gov (NCT01793129).^15^

**Table 2b:** Results of the modified Sarnat neurologic examination

	Total	Infants with fetal acidosis		*P* value
		Yes	No	
No. of infants	102	9 (9)	93 (91)	—
Number of abnormal category of modified Sarnat neurologic examination^a^				
Level of consciousness	8 (8)	5 (56)	3 (3)	<0.01
Spontaneous activity	15 (15)	6 (67)	9 (10)	<0.01
Posture	5 (5)	2 (22)	3 (3)	0.06
Tone	15 (15)	6 (67)	9 (10)	<0.01
Primitive reflexes Suck Moro	33 (32)	7 (78)	26 (28)	<0.01
Autonomic system Pupils Heart rate Respiration	7 (7)	4 (44)	3 (3)	<0.01
Total score^b^	7 ± 3	12 ± 5	7 ± 2	<0.01

^a^Values in parenthesis are percentage of values in the column

^b^Mean ± SD

### Inter-rater reliability

Table 3a, b demonstrates the severity of the neonatal encephalopathy (normal/mild, moderate, severe) as designated by two independent examiners for each category of the neurologic examination. Among 86 newborns, the reliability of the neurologic exam was determined first between GS study investigator and the wider group of attending neonatologists and showed that the inter-rater score, or kappa, was good to excellent (*k* > 0.72) in most categories except for Moro and tone, which showed fair agreement (*k* = 0.51, Table 3a). Disagreement occurred predominantly in assigning a moderate stage when describing incomplete adduction for the Moro and upper extremity hypotonia when assessing for tone.

**Table 3:** Inter-rater agreement of the modified Sarnat neurologic examination performed between (a) gold standard study investigator and neonatology attending physicians and (b) two gold standard examiners in infants of 32–36 weeks’ gestation

3a	Gold standard study investigator			Attending neonatologist			*N* = 86
Modified Sarnat exam category	Normal/mild	Moderate	Severe	Normal/mild	Moderate	Severe	Kappa (95% CI)
LOC	79	4	3	79	4	3	0.84 (0.63, 1.0)
Spontaneous	72	10	4	69	13	4	0.80 (0.64, 0.97)
Activity							
Posture	81	5	0	80	5	1	0.90 (0.71, 1.0)
Tone	74	10	2	59	26	1	0.46 (0.26, 0.66)
Suck	58	23	5	53	28	5	0.72 (0.57, 0.88)
Moro	71	9	6	56	24	6	0.51 (0.32, 0.70)
Pupils	82	3	1	81	4	1	0.88 (0.65, 1.0)
Heart rate	86	0	0	86	0	0	1.0 (1.0,1.0)
Respiration	82	3	1	81	1	4	0.88 (0.65, 1.0)

3b	Gold standard study investigator			Gold standard examiner			*N* = 20
Modified Sarnat exam category	Normal/mild	Moderate	Severe	Normal/mild	Moderate	Severe	Kappa (95% CI)
LOC	16	3	1	16	3	1	1.0 (1.0, 1.0)
Spontaneous	16	3	1	16	3	1	1.0 (1.0, 1.0)
Activity							
Posture	19	1	0	19	1	0	1.0 (1.0, 1.0)
Tone	14	5	1	12	7	1	0.78 (0.49, 1.0)
Suck	15	3	2	16	2	2	0.86 (0.57, 1.0)
Moro	17	0	3	13	4	3	0.49 (0.09, 0.90)
Pupils	19	0	1	19	0	1	1.0 (1.0, 1.0)
Heart rate	20	0	0	20	0	0	1.0 (1.0, 1.0)
Respiration	17	0	3	17	0	3	1.0 (1.0, 1.0)

*CI* confidence interval, *LOC* level of consciousness

In a subset of 20 newborns who were examined by two GS examiners, the inter-rater reliability demonstrates near perfect agreement (*k* > 0.78) except for the Moro category (*k* = 0.49; Table 3b).

In secondary analysis of infants born at 32–34 weeks’ gestation, agreement was poor/fair irrespective of the examiners’ experience for both tone and Moro categories, with kappa ranging from 0.20 to 0.60 (95% confidence interval (CI), 0.0–1.0). In contrast, at 35–36 weeks’ gestation, GS examiners showed perfect agreement (*k* = 1.0) even in the tone and Moro categories. However, when the GS examiner was compared to a wider group of attending examiners, the agreement in the Moro and tone categories was fair, *k* = 0.46 (95% CI, 0.0–0.9).

### Modified Sarnat examination, fetal acidosis, and encephalopathy

Nine infants had fetal acidosis with umbilical cord arterial blood pH ≤ 7.0 or BD ≥ −16. Of the 9 infants with fetal acidosis born at 32–35 weeks, 7 (5.9 per 1000 live births) were diagnosed with HIE.

**Fig. 1:**
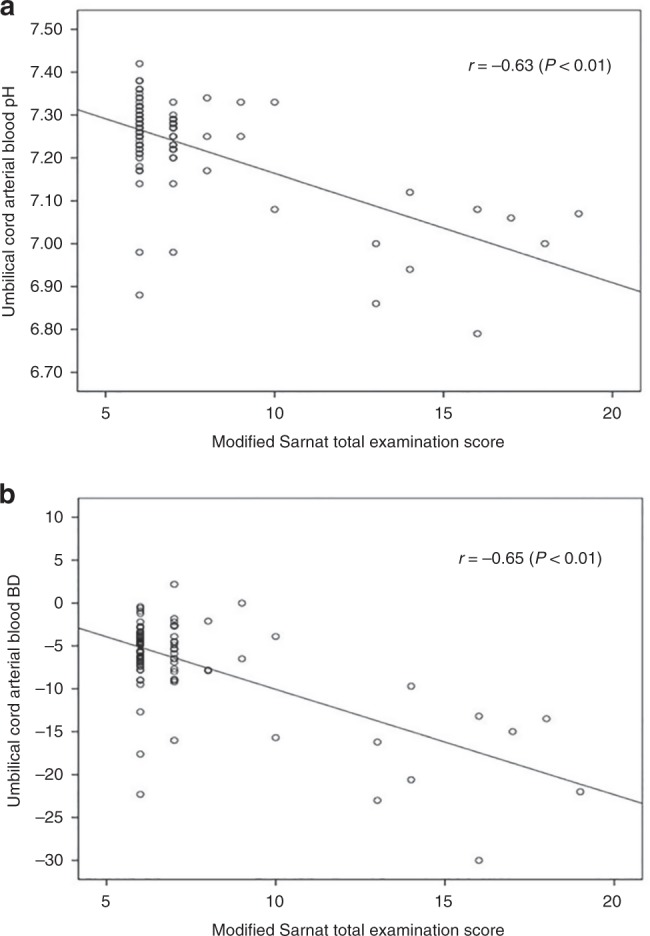
Scatter plots showing the correlation of the total modified Sarnat neurologic examination score performed on 102 infants of 32–36 weeks’ gestation by study investigator in the first 6 h of life, with umbilical cord arterial pH (**a**) and base deficit (**b**). A higher total score correlated with worse acidosis as manifested by lower pH and higher BD, *r* = −0.63 and *r* = −0.65, respectively (*P* < 0.01). *BD* base deficit

Figure 1 shows the significant correlation between increasing abnormalities on the total Sarnat neurologic exam score given by the study investigator, who performed all of the 102 neurologic exams, and metabolic acidosis with umbilical cord pH (*r* = −0.63, Fig. 1a, *P* < 0.01) and BD (*r* = −0.65, Fig. 1b, *P* < 0.01).

## Discussion

This study is the first to test the reliability of the modified Sarnat neurologic examination and correlate abnormalities of the exam with fetal acidosis in infants born at 32–36 weeks’ gestation. Among the most experienced examiners, inter-rater reliability was nearly perfect except for the Moro reflex. When comparing the GS study investigator to the group of neonatologists, reliability was excellent except for the Moro reflex and tone categories. Current study findings support that examiner training and experience was associated with improved reliability in assessment of tone while the Moro reflex is primarily driven by gestational maturity and its assessment is problematic independent of the training and experience of the examiner.

Encephalopathy in infants born at 32–36 weeks’ gestation can result physiologically from a combination of asphyxia and maturational disturbances, and currently there is no validated neurological examination to identify and classify the exam in this specific population. The detailed analysis of encephalopathy as required by the modified Sarnat examination is limited by what can be elicited in a neurologic examination at each level of gestational maturation of the infant.^19^ Late and moderately preterm infants have developmental differences in primitive reflexes, tone, and posture that are controlled by the degree of prematurity and thus can confound the interpretation of the Sarnat score. Flexor posture in the lower extremities is present by 32 weeks’ gestation but only gradually evolves in the upper extremities until present at ≥36 weeks’ gestation.^20^ Similarly, the Moro reflex may be incomplete until 37 weeks’ gestation since the anterior flexion of the upper extremities may be absent in younger gestational age infants. Pupillary reaction to light begins to appear at 30 weeks’ gestation but is not present consistently until approximately 32–35 weeks’ gestation. Findings from this study confirm that the developmentally regulated categories can be difficult to evaluate in the preterm infant.^19–22^

The present study shows that, when using the modified Sarnat score in preterm infants, there is less agreement in the less mature infants of ≤34 weeks’ gestation. This phenomenon was seen irrespective of the experience of the examiners and is consistent with reports using other neurologic scales.^20–23^ In those studies, when compared to term infants, late and moderately preterm infants had less mature performances in several items, including less axial tone with substantial head lag, less flexor tone, and less spontaneous activity. In addition, provider and knowledge-based diversity can contribute to subjectivity when performing the neurological exam.^24^ In order to overcome clarity issues and ensure reliability, examiners should be trained by certified GS examiners who ideally have participated in evidence-based studies utilizing that assessment, as was done in the current study. While the focus on the examination to be performed by trained and certified attending physicians added to the rigor of the study, it is important to note that study findings are likely to overestimate agreement if compared to a clinical setting outside of standardized protocols. Another issue affecting the assessment of reliability is the lower frequency of preterm infants with fetal acidosis necessitating a Sarnat examination. For instance, at our institution the experienced GS examiners screen approximately 50 term infants with fetal acidosis yearly with a neurological examination, which is 10-fold higher than the average of 3–5 per year born with fetal acidosis at 32–35 weeks’ gestation who require such examination.

Moro and tone assessment are included in the majority of neurological assessments for use in this population and are the most used clinically. The findings from this study show that these are the least reliable elements with lower inter-rater agreement, supporting the need for clear and detailed descriptions for scoring criteria of these items. With respect to assessment of tone, agreement was better between the most experienced examiners suggesting that ongoing training and education may aid in the identification of maturational and individual differences. In contrast, agreement in the Moro reflex was poor and no improvement was seen between the experienced study investigator or GS examiners and the group of certified neonatologists. This suggest that the Moro reflex be either removed or simply coded as “present” or “absent” since the intermediate category is developmentally appropriate in late and moderately preterm infants.

The modified Sarnat examination findings in neonates born at 32–35 weeks’ gestation suggest that further research into development of a standardized, gestational age-specific, assessment tool for classification of HIE in these infants is needed. This is clinically pertinent as the developmentally immature preterm infant’s brain appears to be even more vulnerable to hypoxic brain injury and side effects of hypothermia therapies.^4,25^ Despite these concerns, published studies report that 2.4% of late preterm infants <36 weeks gestation are undergoing hypothermia therapy with limited description of the neurologic exam criteria used in these infants.^4,25–31^

The true incidence of hypoxic encephalopathy in premature infants is not accurately known and only a handful of retrospective studies have attempted to estimate its occurrence.^10^ Schmidt and Walsh reported an incidence of 8 per 1000 live births during a 6-year period in a single center, and its diagnosis was based on a 5 min Apgar score <5 with abnormal umbilical cord gas and any neurologic abnormality.^11,12^ In 2012 at PHHS, we retrospectively reported an HIE incidence of 5 per 1000 live births among inborn infants at 33–35 weeks’ gestation.^1^ The current prospective study, which used the blood gas criteria and the modified Sarnat scoring designated for term newborns,^7^ confirmed an HIE incidence of 5.9 per 1000 live births in neonates <35 weeks’ gestational age.

A strength of this study is the participation of a large number of clinicians in a single unit and that the independent standardized evaluations were concomitantly performed by certified attending neonatologists and GS examiners within the first 6 h of age. Other strengths include a prospective design, large sample size with predefined analysis and power calculations, and that umbilical cord blood gas analysis was performed routinely on all infants.

We acknowledge the study has several limitations. First, the reliability coefficients can be influenced by the prevalence of hypoxic encephalopathy which was low in the study cohort of preterm infants. However, the inclusion of low-risk infants was necessary in order to test the reliability of the examination and avoid any bias by blinding of examiners to the umbilical cord gas results. Second, although training and certification were standardized to ensure study rigor, there was no formal tracking of the examiners’ aptitude before the study. Lastly, the agreement in this study is likely overestimated when compared to general clinical settings of busy diverse intensive care or transport settings with many providers further emphasizing the need of standardized training.

## Conclusion and future directions

The optimal examination for determination of acute encephalopathy in preterm infants is not known. An ideal form should be easy to perform, reliable and reproducible between examiners in real time and by video or telemedicine evaluations, and accurately predict outcome. Standardization of care and use of simple, quantitative clinical assessments to aid in prognosis and medical management is important and necessary.

In the current modified Sarnat form adapted from term infants and used in infants at 32–35 weeks’ gestation, the evaluation of tone was improved by examiners’ experience, while the Moro reflex was primarily dependent on gestational age. The current HIE forms designed for term infants and extrapolated in practice for preterm infant may not be optimal. At our institution, we have adopted a two-prong approach: (1) education targeting the assessment of tone in preterm infants and (2) studies testing a new neurological examination form omitting the Moro and detailing evaluations of the tone adapted from the Dubowitz and Hammersmith Infant Neurologic Examinations.^22,24^ These ongoing studies could aid in the improved assessment of the hypoxic encephalopathy in premature neonates.

## References

[1] Chalak LF (2012). Perinatal acidosis and hypoxic-ischemic encephalopathy in preterm infants of 33 to 35 weeks’ gestation. J. Pediatr..

[2] Volpe JJ (1979). Value of the neonatal neurologic examination. Pediatrics.

[3] Gopagondanahalli KR (2016). Preterm hypoxic–ischemic encephalopathy. Pediatrics.

[4] Herrera TI (2018). Outcomes of preterm infants treated with hypothermia for hypoxic-ischemic encephalopathy. Early Hum. Dev..

[5] Amiel-Tison C (1968). Neurological evaluation of the maturity of newborn infants. Arch. Dis. Child..

[6] Sarnat HB, Sarnat MS (1976). Neonatal encephalopathy following fetal distress. A clinical and electroencephalographic study. Arch. Neurol..

[7] Shankaran S (2005). Whole-body hypothermia for neonates with hypoxic-ischemic encephalopathy. N. Engl. J. Med..

[8] Volpe JJ (2009). The encephalopathy of prematurity–brain injury and impaired brain development inextricably intertwined. Semin. Pediatr. Neurol..

[9] Einspieler C, Prechtl H, Ferrari F, Cioni G, Bos A (1997). The qualitative assessment of general movements in preterm, term and young infants – review of the methodology. Early Hum. Dev..

[10] Salhab WA, Perlman JM (2005). Severe fetal acidemia and subsequent neonatal encephalopathy in the larger premature infant. Pediatr. Neurol..

[11] Schmidt JW, Walsh W (2010). Hypoxic-ischemic encephalopathy in preterm infants. J. Neonatal Perinat. Med..

[12] Walsh WF, Butler D, Schmidt JW (2015). Report of a pilot study of cooling four preterm infants 32-35 weeks gestation with HIE. J. Neonatal Perinat. Med..

[13] Romeo DM (2017). Neonatal neurological examination during the first 6h after birth. Early Hum. Dev..

[14] Fenichel GM (1993). Neurological examination of the newborn. Brain Dev..

[15] Preemie Hypothermia for Neonatal Encephalopathy. ClinicalTrials. gov Identifier: https://ClinicalTrials.gov/show/NCT01793129; 2018.

[16] Prempunpong C (2017). Prospective research on infants with mild encephalopathy: the PRIME study. J. Perinatol..

[17] American College of Obstetricians and Gynecologists, American Academy of Pediatrics. *Neonatal Encephalopathy and Neurologic Outcome*, 2nd edn (American College of Obstetrics and Gynecologists, Washington, DC, 2014).

[18] Cicchetti DV (1994). Guidelines, criteria, and rules of thumb for evaluating normed and standardized assessment instruments in psychology. Psychol. Assess..

[19] Murray DM (2010). The predictive value of early neurological examination in neonatal HIE and neurodevelopmental outcome at 24 months. Dev. Med. Child Neurol..

[20] Chalak LF, Rouse DJ (2011). Neuroprotective approaches: before and after delivery. Clin. Perinatol..

[21] Brazelton, T. B. *Neonatal Behavioural Assessment Scale Clinics in Developmental Medicine No. 50. London: Spastics International Medical Publications/William Heinemann Medical Books* (JB Lippincott Co, Philadelphia, 1973).

[22] Dubowitz L, Mercuri E, Dubowitz V (1998). An optimality score for the neurologic examination of the term newborn. J. Pediatr..

[23] Romeo DM (2013). Neonatal neurological examination of late preterm babies. Early Hum. Dev..

[24] Maitre NL, Chorna O, Romeo DM, Guzzeta A (2016). Implementation of the Hammersmith Infant Neurological Exam in a high-risk infant follow-up program. Pediatr. Neurol..

[25] Gunn AJ, Bennet L (2008). Brain cooling for preterm infants. Clin. Perinatol..

[26] Pfister RH (2011). Hypothermia in practice, initial observations from the Vermont Oxford Network. PAS Abstr..

[27] Higgins RD, Shankaran S (2011). Hypothermia: novel approaches for premature infants. Early Hum. Dev..

[28] Azzopardi A (2000). Pilot study of treatment with whole body hypothermia for neonatal encephalopathy. Pediatrics.

[29] Zanelli SA, Naylor M, Dobbins N, Quigg M, Goodkin HP (2008). Implementation of a “Hypothermia for HIE” program: 2 year experience in a single NICU. J. Perinatol..

[30] Kilani RA (2002). The safety and practicality of selective head cooling in asphyxiated human newborn infants, a retrospective study. J. Med. Liban..

[31] Rao R (2017). Safety and short-term outcomes of therapeutic hypothermia in preterm neonates 34-35 weeks gestational age with hypoxic-ischemic encephalopathy. J. Pediatr..

